# The long non‐coding RNA *PVT1* represses *ANGPTL4* transcription through binding with EZH2 in trophoblast cell

**DOI:** 10.1111/jcmm.13405

**Published:** 2017-11-29

**Authors:** Yetao Xu, Yifan Lian, Yuanyuan Zhang, Shiyun Huang, Qing Zuo, Nana Yang, Yanzi Chen, Dan Wu, Lizhou Sun

**Affiliations:** ^1^ Department of Obstetrics and Gynecology The First Affiliated Hospital of Nanjing Medical University Nanjing Jiangsu China; ^2^ Department of Gastroenterology Zhongshan Hospital Xiamen University Xiamen Fujian China; ^3^ Department of Emergency The First Affiliated Hospital of Nanjing Medical University Nanjing Jiangsu China

**Keywords:** preeclampsia, *PVT1*, PRC2, proliferation

## Abstract

Despite progress in diagnostics and treatment for preeclampsia, it remains the foremost cause of maternal and foetal perinatal morbidity and mortality worldwide. Over recent years, various lines of evidence have emphasized long non‐coding RNAs (lncRNAs) which function as an innovative regulator of biological behaviour, as exemplified by proliferation, apoptosis and metastasis. However, the role of lncRNAs has not been well described in preeclampsia. Here, we identified a lncRNA,*PVT1*, whose expression was down‐regulated in qRT‐PCR analyses in severe preeclampsia. The effects of *PVT1* on development were studied after suppression and overexpression of *PVT1* in HTR‐8/SVneo and JEG3 cells. *PVT1* knockdown notably inhibited cell proliferation and stimulated cell cycle accumulation and apoptosis. Exogenous *PVT1* significantly increased cell proliferation. Based on analysis of RNAseq data, we found that *PVT1* could affect the expression of numerous genes, and then investigated the function and regulatory mechanism of *PVT1* in trophoblast cells. Further mechanistic analyses implied that the action of *PVT1* is moderately attributable to its repression of *ANGPTL4 via* association with the epigenetic repressor Ezh2. Altogether, our study suggests that *PVT1* could play an essential role in preeclampsia progression and probably acts as a latent therapeutic marker; thus, it might be a useful prognostic marker when evaluating new therapies for patients with preeclampsia.

## Background

Preeclampsia (PE) is a multisystem syndrome of human gestation that is characterized by hypertension, proteinuria and oedema after 20 weeks of pregnancy. New guidelines from the American College of Obstetrics and Gynecology (ACOG) state that PE can also be diagnosed in the non‐existence of proteinuria in hypertensive women complicated with pulmonary oedema, impaired liver function, thrombocytopenia and associated renal insufficiency, and visual disorders 1. PE is the main reason for maternal and foetal perinatal morbidity and mortality worldwide 2, 3, affecting 3–5% of pregnancies and causing approximately 60,000 maternal deaths annually 4. Delivery is the only cure for PE 5, and effective prevention strategies have not yet been developed.

PE originates from the placenta, and its sequelae also affect multiple organ systems. Many factors are associated with the etiopathogenesis of PE, including endothelial dysfunction 6, inflammatory cytokines 7, 8, 9, oxidative stress 10, 11, imbalances between pro‐angiogenic and antiangiogenic factors 12, 13, and dietary and genetic factors 14. Among these factors, endothelial dysfunction is the common trigger of most medical symptoms and could lead to hypertension, oedema, proteinuria and other symptoms 15. During the early development of the placenta, extravillous trophoblasts (EVTs) originating from the foetus can migrate and invade the maternal spiral arteries of the myometrium and/or decidua, providing a substitute for the endothelial layers of the uterine spiral arteries. This process, known as maternal spiral artery remodelling, is vital for reducing blood flow resistance and/or aggregating uteroplacental perfusion 16. In PE, this process is impaired 17, 18. Owing to increased apoptosis 19, 20, reduced EVT proliferation 21, and impaired EVT migration and invasion capacity 22, EVTs are not able to effectively invade the spiral arteries in the myometrium in patients with PE. However, despite extensive research, the specific mechanisms related to the progression of cell proliferation, apoptosis, invasion and migration in preeclampsia remain uncertain.

Recent studies have elucidated the genetic events that play essential roles in the progression and/or improvement of Preeclampsia. Non‐coding RNAs have been revealed to have several principal regulatory functions in transcription and post‐transcriptional processes 23. In particular, abnormal regulation of lncRNAs, which are longer than 200 bop and do not encode proteins, is associated with numerous human disorders 24, including cardiovascular disease 25 and neurodegenerative diseases 26. Moreover, lncRNAs play fundamental roles in both normal development and disease 26, and lncRNAs are correlated with diverse cellular processes, including stem cell pluripotency, apoptosis, proliferation, migration and invasion 27, 28. We have identified two lncRNAs that participate in the occurrence and progress of PE in our previous study 29, 30. However, most lncRNAs have not been shown to regulate the associated functions and mechanisms of trophoblasts or to be involved in PE. Accordingly, identifying PE‐associated lncRNAs and related molecular mechanisms is essential for understanding the progress of preeclampsia and establishing improved treatment strategies.

Accordingly, in this study, we assessed the relative expression and role of the lncRNA *PVT1* in placental tissues from women with normal pregnancies and PE. In addition, we further explored the effects of *PVT1* on trophoblast cell proliferation, apoptosis, migration and invasion *in vitro*. Our results provide important insights into the role of *PVT1* in the aberrant characteristics of trophoblasts in PE and may act as a potential biomarker for preeclampsia diagnosis and therapy.

## Materials and methods

### Tissue samples and patients

We obtained 52 paired placental tissues from normal pregnancies and preeclampsia women, who underwent caesarean deliveries in Jiangsu Province Hospital from 2015 to 2016, then all obtained tissue samples were instantaneously stored at −80°C before RNA and protein extraction. And the clinicopathological characteristic of the normal pregnancies and pre‐eclamptic women has been recapitulated in Table 1. And this research was authorized by the Ethnics Board of the First Affiliated Hospital of Nanjing Medical University, China. Relative written informed agreements were gotten from patients which meet the criteria were included in this study.

**Table 1:** Clinical characteristics of pre‐eclamptic and normal pregnancies

Variable	PE (*N* = 52)	Normal (*N* = 52)	*P* value* Normal *versus P*
Maternal age (year)	33.96 ± 5.639	34.69 ± 3.226	*P* > 0.05
Maternal weight (kg)	74.75 ± 10.885	72.28 ± 9.185	*P* > 0.05
Smoking	0	0	*P* > 0.05
Systolic blood pressure (mm Hg)	162.51 ± 15.472	116.73 ± 7.728	*P* < 0.01
Diastolic blood pressure (mm Hg)	106.71 ± 11.155	74.59 ± 7.57	*P* < 0.01
Proteinuria (g/day)	>0.3 g	<0.3 g	*P* < 0.05
Body weight of infant (g)	2365.57 ± 1013.032	3389.42 ± 387.72	*P* < 0.05

All results are presented as mean ± S.D. S.D., standard deviation. Obtained by one‐way analysis of variance using SPSS 18.0 software (SPSS Inc, Chicago, IL, USA). (Values are mean ± SD; *: *P* < 0.05; **: *P* < 0.01).

### Cell culture

Trophoblast Cells HTR/SVneo, which derived from primary extra villous trophoblast cell, was friendly offered by Dr. Charies Graham, Queen's University, Canada. And HTR/SVneo was cultured in RPMI 1640 (GIBCO, Nanjing, China), which added with 5% FBS (GIBCO, Invitrogen, Carlsbad, CA, USA),100 U/ml penicillin and 100 mg/ml streptomycin (Invitrogen) in humidified air at 37C with 5% CO_2_. Other cell lines, as exemplified by JEG‐3, BeWo, Wish and HUVEC‐C, were all purchased from the Chinese Academy of Sciences Committee (Shanghai, China).

### Transfection of cell lines

Generally, HTR/SVneo and JEG3 were cultivated at six‐well plates until confluent and then transfected with corresponding siRNAs(10 ul) or scrambled negative control siRNA(10 ul) or plasmid vectors(4 ug) by exploiting Lipofectamine 2000 (Invitrogen). Plasmid vectors (pcDNA3.1‐*PVT1*, and pcDNA) were distilled by DNA Midiprep kit (Qiagen, Hilden, Germany). The nucleotide sequences of siRNAs for *PVT1*,* EZH2* and scramble negative control(si‐NC) purchased from Invitrogen and have been summarized in Table S2. To ectopically express the *PVT1*, the full‐length complementary DNA of *PVT1* was synthesized by Realgene (Nanjing, China) and subcloned into the pcDNA3.1(+) vector (Invitrogen), on the basis of the manufacturer's instructions. At 36‐hr post‐transfection, cells would be harvested for further studies, such as qRT‐PCR and Western blot analysis.

### RNA preparation and qRT‐PCR assays

Actually, the total RNAs were isolated from clinic sample tissues or cultured cells using TRizol reagent (Invitrogen), following the manual. This experiment was executed in accordance with the relevant guidelines. 1 ug RNA was reverse transcribed to cDNA in a final volume of 20 ul with PrimerScript RT Master Mix (Takara, Dalian, China). Then we applied the SYBR Premix Ex Taq (Takara, Dalian, China) to determine *PVT1* levels, on the basis of the manufacturer's protocol. The resulting data were normalized to the expression of *GAPDH*. The relevant primers were listed in Table S1. qRT‐PCR assays were implemented on an ABI 7500, and our results were calculated and expressed relative to threshold cycle (shown as ΔCT) values, then converted to fold changes. This experiment was performed in accordance with the relevant guidelines and regulations.

### Subcellular fractionation location

The isolation of the nuclear and cytosolic fractions was implemented by exploiting the PARIS Kit (Life Technologies, Carlsbad, CA, USA), following the manual.

### Cell proliferation assays

Cell viability was supervised with the MTT (Cell Proliferation Reagent Kit I) Penzberg, Germany). The HTR/SVneo and JEG‐3 were treated with si‐*PVT1* or pcDNA‐*PVT1*(3500 cells/well) and were cultivated in 96‐well plates with five duplicate. Cell viability was seasoned every 24 hrs according to the manual. The absorbance was detected at 490 nm with an ELx‐800 University Microplate Reader (BioTek, Winooski, VT, USA).

### Ethynyl deoxyuridine analysis (Edu)

As we know, the Edu assay was implemented as a complementary method to authenticate the proliferation level more accurately than MTT and colony formation assays. Usually, we exploit the 5‐ethynyl‐2‐deoxyuridine labelling/detection kit (Ribobio, Guangzhou, China) to evaluate cell proliferation, following the manufacturer's recommendation.

### Flow cytometric analysis of cell cycle and apoptosis

These trophoblast cell lines, treated with si‐*PVT1*‐1# and si‐*PVT1*‐2#, were harvested 36 hrs after transfection, respectively and washed twice with cold PBS. Then, we exploit FITC‐Annexin V and Propidium iodide (PI) using the FITC‐Annexin V Apoptosis Detection Kit (BD Biosciences Franklin Lakes, NJ, USA) following the manufacturer's manual. Cell cycle analysis was stained with propidium oxide by the Cycle TEST PLUS DNA Reagent Kit (BD Biosciences) according to the protocol and analysed by FACScan. The ratio of the cells in G0/G1, S and G2/M phase was calculated and compared.

### Cell migration and invasion assays

The ability of cell migration and invasion were analysed by transwell assays as previously reported in Zuo *et al*. 31.

### Western blot assay

WB assays were implemented to detected the relative level of protein as formerly reported in Zuo *et al*. 31, and incubated with specific antibodies (*GAPDH*,* EZH2*,* ANGPTL4* purchased from CST or Proteintech) at 1:1000 concentration. The intensity of autoradiogram protein bands was quantified by the Equipment of Quantity One software (Bio‐Rad Hercules, CA, USA).

### RNA‐seq bioinformatic analysis

The RNA‐Seq experiments were implemented by Beijing Genomics Institute (Beijing, China). mRNA‐seq library was arranged for exploiting standard Illumina manuals. Briefly, RNAs were obtained from si‐NC, or si‐*PVT1*‐1# transfected HTR‐8/SVneo cells, were isolated utilizing TRIzol reagent (Invitrogen). mRNA extraction was executed using Dynabeadsoligo (Invitrogen Dynal). To stablish the mRNA‐seq library, the cDNAs were next fragmented *via* nebulization and the standard Illumina protocol followed.

### RNA immunoprecipitation assays (RIP)

RIP experiments were performed as formerly reported in Zuo *et al*. 31, following the manufacturer's instructions. EZH2 and SUZ12 antibodies used for IP were from Millipore (Billerica, MA, USA). At the end of RIP experiment, the obtained RNA was subjected to qRT‐PCR analysis to demonstrate the presence of *PVT1* and IgG using specific primers which have been listed in Table S2.

### Chromatin immunoprecipitation assays (ChIP)

We performed ChIP assays with the EZ‐ChIP KIT according to the manual (Millipore). *EZH2*,* SUZ12* and H3K27me3‐specific antibodies were purchased from Millipore or mIgG/gIgG as negative control. Precipitated chromatin DNA was recovered and subjected to qRT‐PCR analysis. The primer sequences were enrolled Table S2.

### Statistical analysis

Student's *t*‐test was utilized to analyse data *in vitro* using SPSS 17.0 statistical software (IBM, Chicago, IL, USA). Furthermore, *P*‐values of less than 0.05 were contemplated statistically significant. These resulting data are recounted as the mean ± S.D. *P* < 0.05(*) or *P* < 0.01(**). At least three times for each experiment which was repeated independently.

## Result

### *PVT1* expression in placental tissues from women with normal pregnancy and severe PE

**Figure 1:**
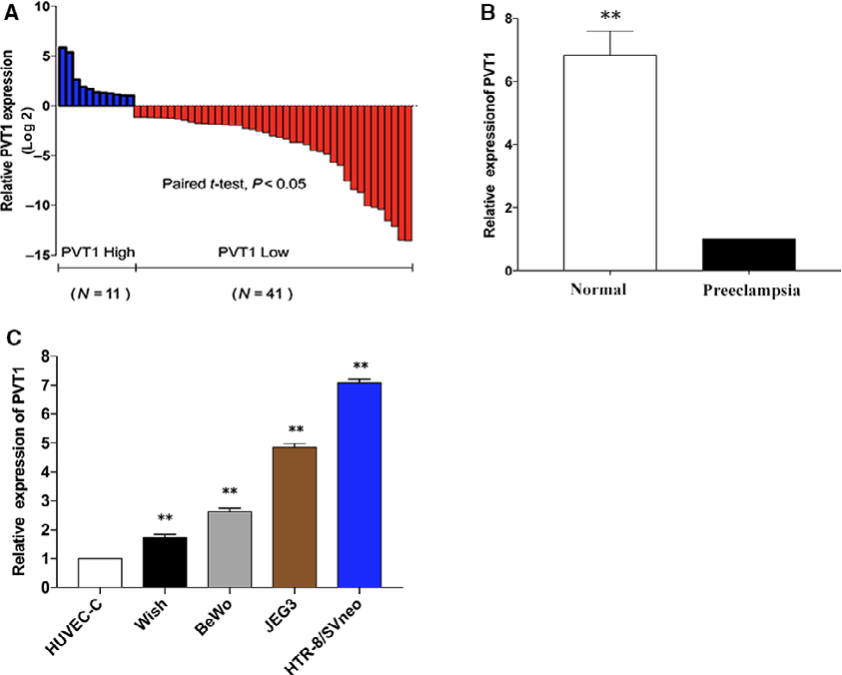
Relative *PVT1* expression in preeclampsia. (**A** and **B**) LncRNA *PVT1* levels are lessened in PE placentas. The expression of *PVT1* was significantly lower in PE samples (*n* = 52) than that in normal placentas (log2). (**C**) The *PVT1* expression in BeWo, WISH, JEG‐3 and HTR‐8/SVneo was normalized to that in HUVEC‐C. At least three times of biological replicates have been performed and presented. *P < 0.05, **P < 0.01.

To ascertain whether *PVT1* was differentially expressed in placental samples from severe preeclampsia women, we quantified *PVT1* expression by qRT‐PCR in 52 paired clinic placental samples from women with normal pregnancies and severe PE. The results showed that *PVT1* expression was lower in pregnant women with PE than that in women with normal pregnancies (Fig. 1A and B). Table 1 illustrates the clinical characteristics of these patients.

### *PVT1* promote cell proliferation and apoptosis in trophoblast cells

As human lncRNAs play indispensable roles in numerous biological behaviour, we detected the expression of *PVT1* in several trophoblast cell lines, including HTR‐8/SVneo, BeWo, and JEG‐3, WISH and HUVEC‐C. As shown in Figure 1C, we discovered that the relative *PVT1* level in this two cell lines was higher compared to WISH, HUVEC‐C and BeWo. Then we examined whether *PVT1* was functionally involved in PE using two different *PVT1*‐specific siRNAs to down‐regulate endogenous *PVT1* expression in this two cell lines. qPCR analysis indicated that *PVT1* expression was sufficiently silenced after treated with specific siRNAs (Fig. S1A); therefore, si‐*PVT1*‐1# and/or si‐*PVT1*‐2# were used for subsequent experiments. Conversely, ectopic overexpression of *PVT1* was successfully induced by transfecting these two cells with a pcDNA 3.1‐*PVT1* expression vector (Fig. S1A).

**Figure 2:**
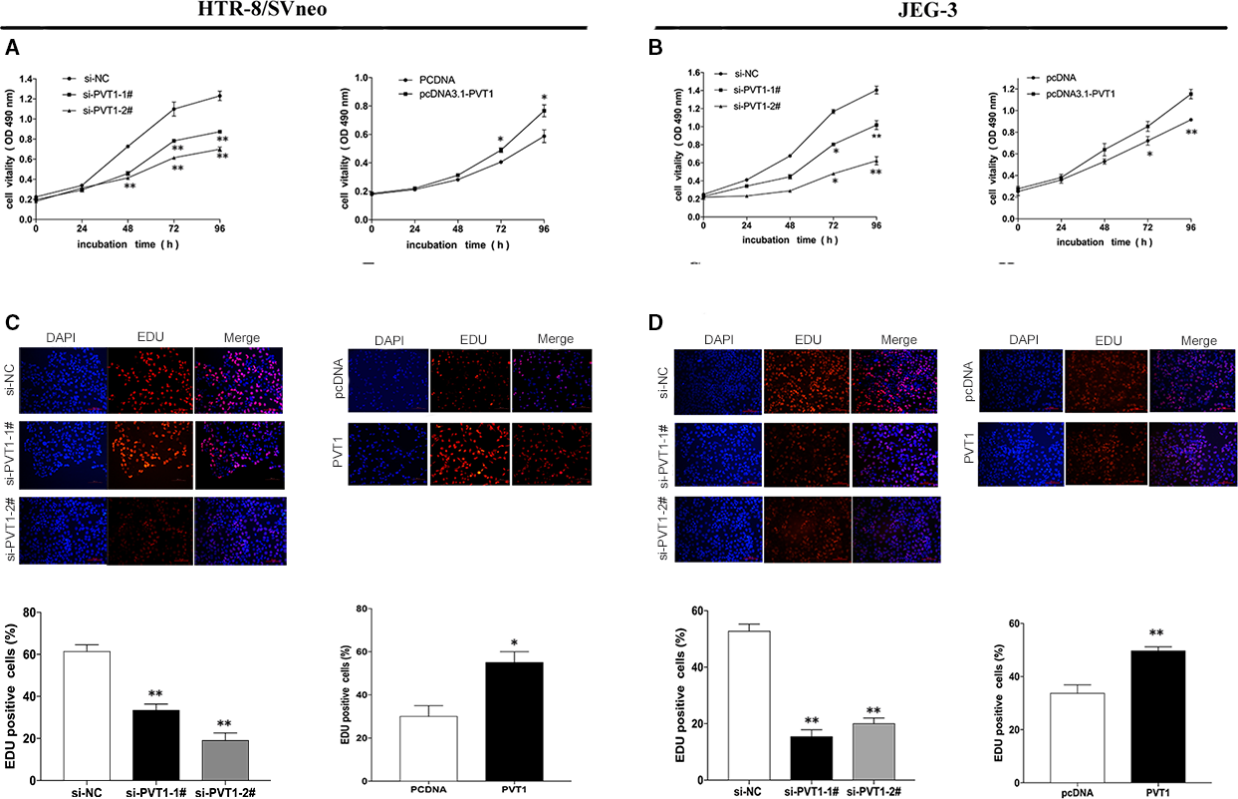
*PVT1* promotes Cells proliferation *in vitro*. (**A** and **B**) MTT assays were implemented to detect the viability of si‐*PVT1*‐1#‐treated or pcDNA3.1‐*PVT1*‐treated Trophoblast Cells. The cell viability after transfected with si‐*PVT1* was significantly lower than that treated with the control. (**C** and **D**). Proliferating Trophoblast Cells were labelled using Edu. The Click‐it reaction shown Edu staining (red). Cell nuclei were stained with DAPI (blue). *P < 0.05, **P < 0.01.

MTT assays showed that knocking down *PVT1* levels significantly inhibited the growth of HTR/SVneo and JEG‐3 cells. Additionally, *PVT1* overexpression promoted cell proliferation in trophoblast cells (Fig. 2A and B). Similarly, EdU/DAPI immunostaining further confirmed these results (Fig. 2C and D). Taken together, these findings supported the positive role of *PVT1* in HTR/SVneo and JEG‐3 cell proliferation, a crucial process in the progression of PE.

**Figure 3:**
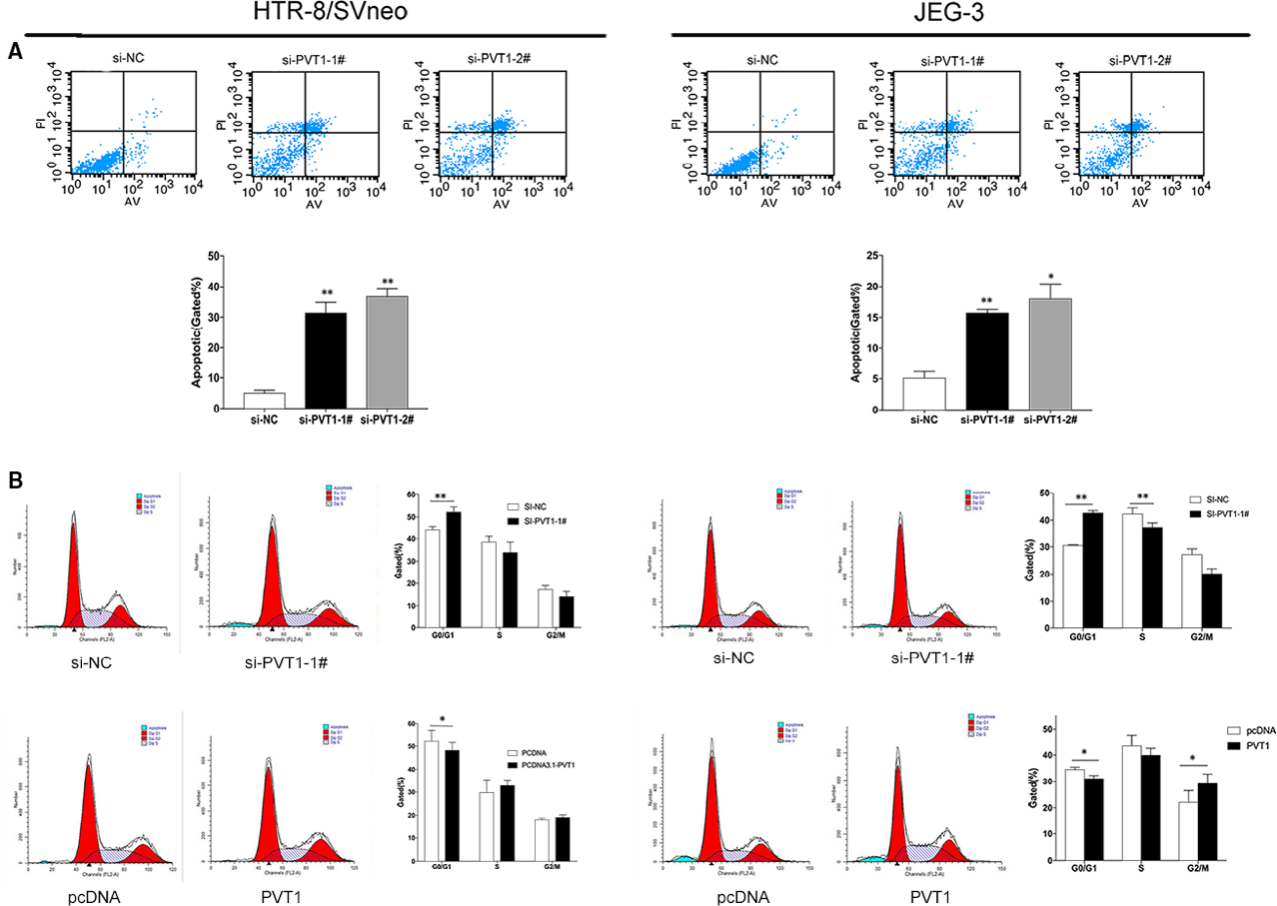
Effect of *PVT1* on apoptosis and cycle in Trophoblast cells. Trophoblast Cells were treated with siRNAs or pcDNA3.1‐*PVT1*. (**A**) The apoptotic rates of cells were measured by Flow cytometry. LL, dead cells; UL, viable cells; LR, early apoptotic cells; UR, terminal apoptotic cells. (**B**) Cell cycle analyses by Flow cytometry *in vitro*. *P < 0.05, **P < 0.01.

Next, we used flow cytometry analysis to explore the mechanisms through which *PVT1* affected the proliferation of trophoblasts cells. The results revealed that knockdown of *PVT1* noticeably induced apoptosis in HTR/SVneo and JEG‐3 trophoblasts compared with that in control cells (Fig. 3A). Moreover, cell cycle progression was meaningfully accumulated at G_0_/G_1_ phase in trophoblasts cells transfected with si‐*PVT1*‐1# compared with that in cells treated with scramble. Conversely, overexpression *PVT1* reduced cell cycle accumulation in G_0_/G_1_ phase (Fig. 3B).

### Effects of *PVT1* on the migration and invasion of HTR/SVneo and JEG‐3 trophoblasts

**Figure 4:**
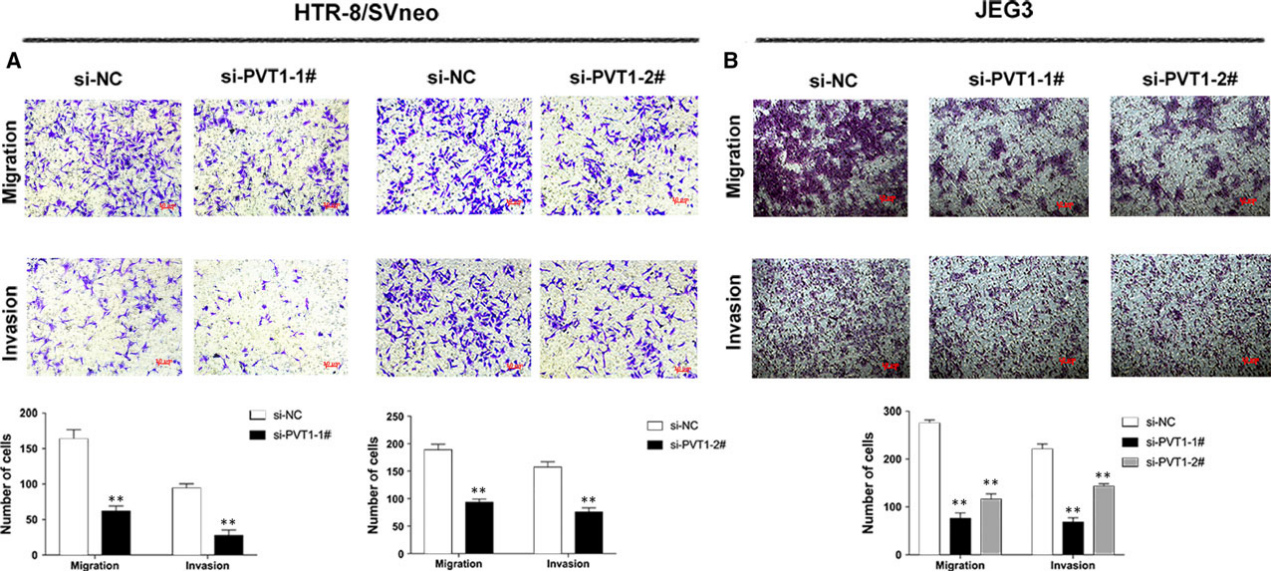
Effect of *PVT1* on Trophoblast cell migration and invasion *in vitro*. (**A** and **B**) Transwell assays were performed to detect the role of *PVT1* on trophoblast cell migration and invasion *in vitro*. The migration and invasion viability of the trophoblast cell transfected with si‐*PVT1* were meaningfully lower than that of the control. The cells on the lower chamber were stained by crystal violet and displayed, and cell number in random five perspectives was analysed. *P < 0.05; **P < 0.01.

The migration and invasion of trophoblasts cells are critical for diseases progression; therefore, we next evaluated the effects of *PVT1* on HTR‐8/SVneo and JEG‐3 cell migration and invasion using transwell assays. The results showed that decreased *PVT1* expression blocked cell migration and invasion compared with that in the control cells (Fig. 4A and B). These results implied that knockdown *PVT1* repressed the migration and invasion of trophoblasts cells.

### Gene expression profiling

**Figure 5:**
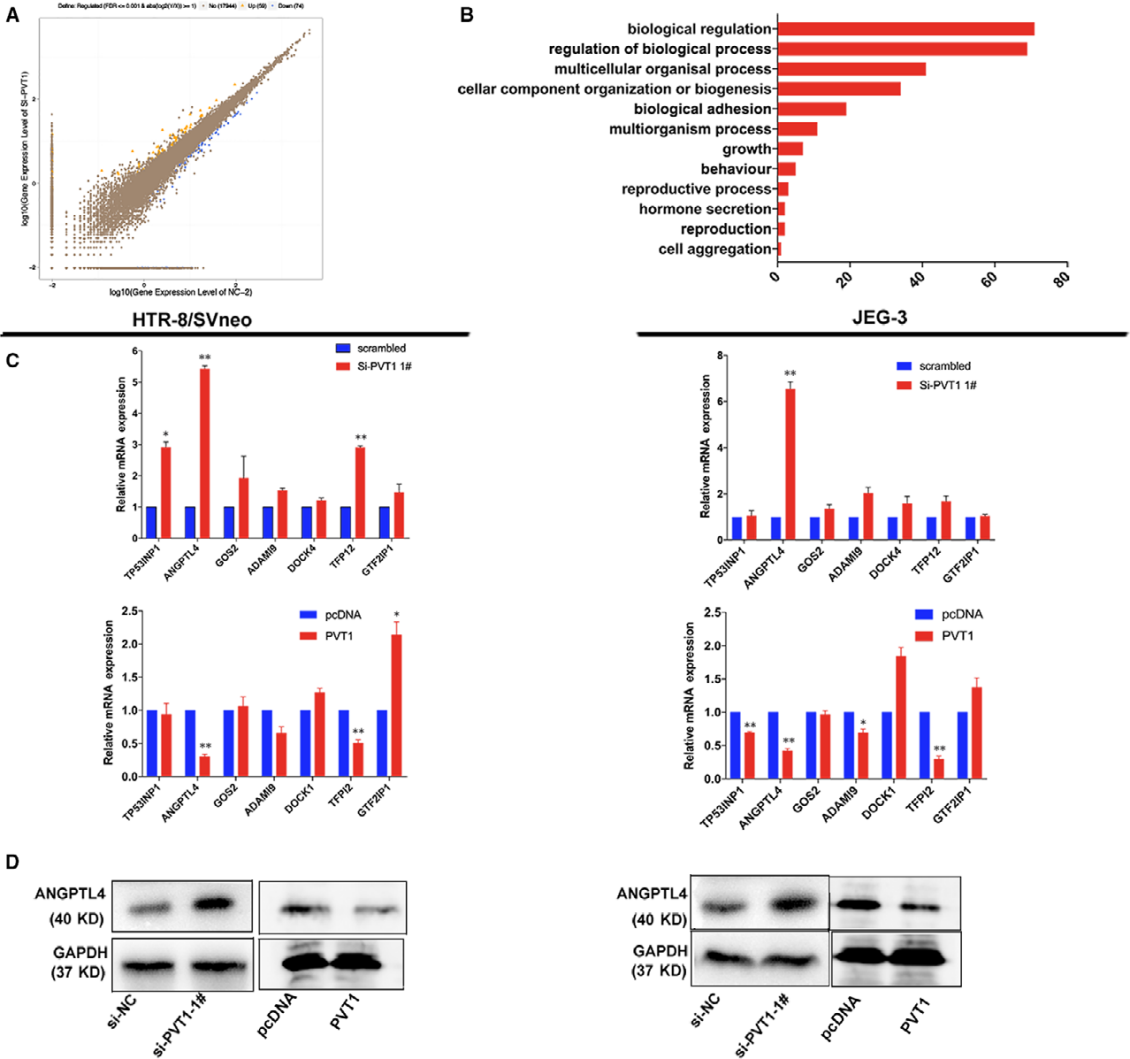
*PVT1* knockdown increases genes expression which contributed to the cell proliferation and migration. (**A**) RNA transcriptome sequencing analysis was performed to analyse gene expression profiling in HTR‐8/SVneo cells following *PVT1* knockdown. The picture showed the all of different expressed gene. (**B**) GO analysis for these genes with abnormal levels between the treating with si‐NC or si‐PVT1 in trophoblast cells. (**C** and **D**) qRT‐PCR analysis reveal abnormal mRNA expression of genes contributed to cell proliferation and migration in trophoblast cells after knockdown or overexpression of *PVT1*. (**E**) WB assay of *ANGPTL4* level after transfecting with si‐*PVT1* or Plasmid vectors (pcDNA3.1‐*PVT1*) into trophoblast cells. *P < 0.05, **P < 0.01.

To elucidate *PVT1*‐associated changes in gene expression, we implemented RNA transcriptome sequencing using control and *PVT1*‐depleted HTR/SVneo cells. A total of 59 mRNAs presented at least a twofold increase in abundance, whereas 74 mRNAs exhibited a twofold or greater reduction in abundance in HTR/SVneo cells after depletion of *PVT1* (Fig. 5A; Table S1). Evaluation of pathways activated by *PVT1* using GO and KEGG databases showed that cell growth was altered in *PVT1*‐depleted cells (Fig. 5B). Additionally, using qRT‐PCR, we verified changes in the expression of representative genes involved in cell growth in HTR‐8/SVneo and JEG‐3 cells. Among transcripts that were related to cell growth, apoptosis, and migration; *E*ncoding angiopoietin‐like 4 (*ANGPTL4*) was significantly up‐regulated/after *PVT1* knockdown (Fig. 5C). On the contrary, it was down‐regulated after overexpression of *PVT1*. Western blotting was also performed and further demonstrated that the *ANGPTL4* proteins were significantly increased/decreased in *PVT1*‐depleted/*PVT1*‐overexpression cells (Fig. 5D).

### *PVT1* epigenetically silenced *ANGPTL4* transcription by binding to EZH2

**Figure 6:**
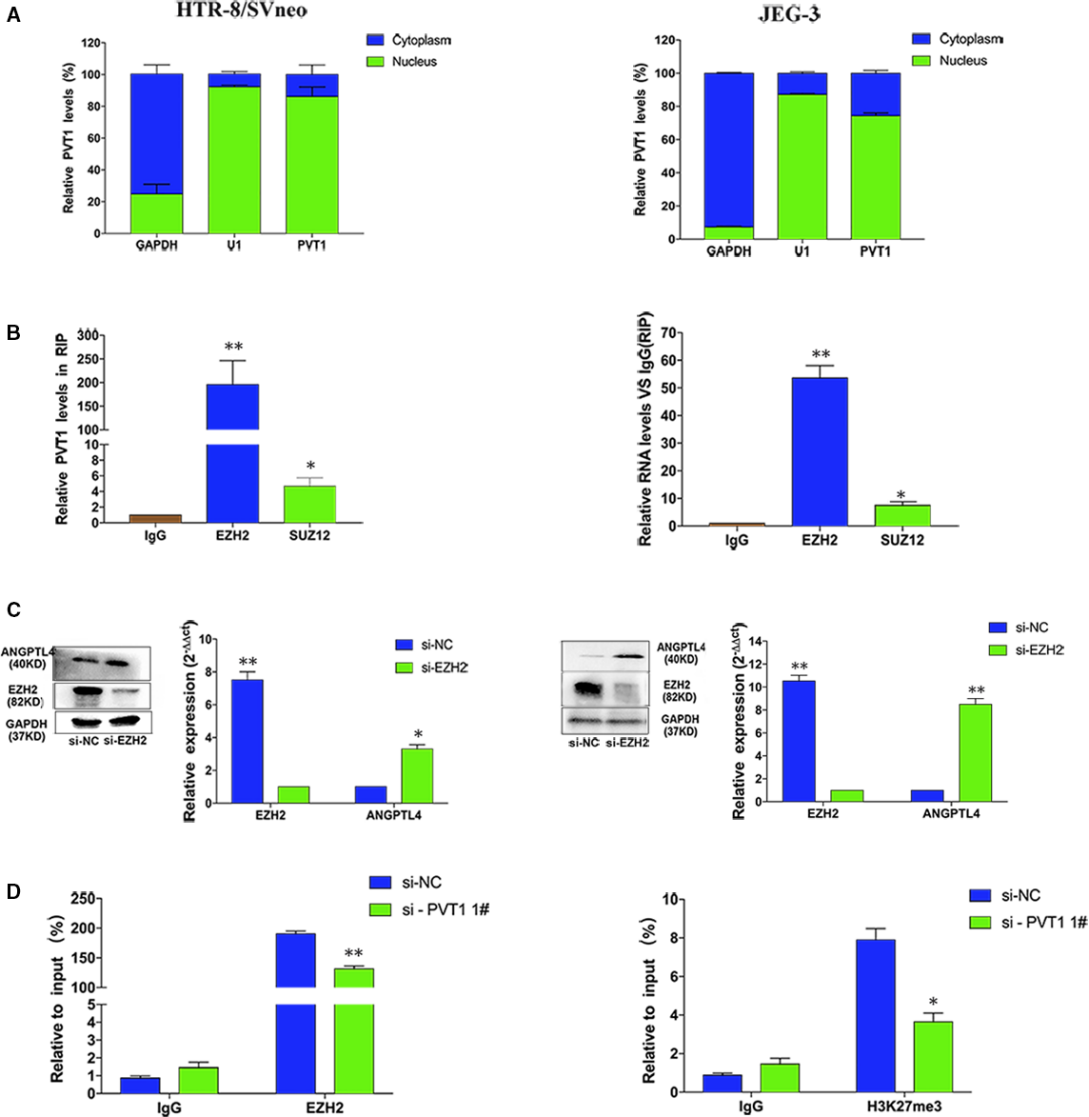
*PVT1* binds to Ezh2 to suppress *ANGPTL4* expression. (**A**) Using qPCR, relative *PVT1* levels are mostly located in nucleus, in which GAPDH and U1 acted as the marker of cytoplasm and nucleus, respectively. (**B**) RIP assays established that *PVT1* could interact with Ezh2. (**C**) Knockdown EZH2 triggered *ANGPTL4* expression at the mRNA levels by qPCR and protein levels by WB. (**D**) ChIP assays uncovered that Ezh2 and H3K27me3 were enriched in the promoter region of *ANGPTL4*, and this enrichment was reduced after *PVT1* knockdown. *P < 0.05, **P < 0.01.

The subcellular location of a molecule may provide insights into its molecular mechanism. Therefore, we evaluated the localization of *PVT1* in nuclear and cytoplasmic fractions in HTR/SVneo and JEG‐3 cells using U1 (RNU1) and GAPDH as markers of the nucleus and cytoplasm, respectively (Fig. 6A). Notably, *PVT1* RNA was predominantly located in the nucleus in HTR/SVneo and JEG‐3 cells. Accordingly, *PVT1* may have functions in transcriptional regulation.

Previous studies have shown that have covered that nearly 20% of human lncRNAs can bind to PRC2 which contained *EZH2*,* SUZ12* and embryonic ectoderm development [EED] in various cells and exert their regulatory functions through interacting with RNA‐binding proteins (RBPs), particularly PRC2 32, 33. Thus, we hypothesized that *PVT1* might modulate *ANGPTL4* expression by recruiting PRC2 in HTR/SVneo and JEG‐3 cells. Indeed, RNA immunoprecipitation (RIP) showed sufficient enrichment of *PVT1* by anti‐*EZH2* antibodies compared with that of the nonspecific IgG control (Fig. 6B).

We further explored the functional relevance of the relationship between *PVT1* and *EZH2*. Repression of *EZH2* expression by siRNAs showed that *ANGPTL4* was up‐regulated (Fig. 6C). Additionally, because PRC2 is known to regulate transcription through histone modification, H3K27me3 in the promoter region of target genes 34, 35 we speculated that *PVT1* may modulate *ANGPTL4* through epigenetic mechanisms in trophoblasts cells. Using ChIP assays with antibodies against *EZH2* and H3K27me3, we analysed that Ezh2 and H3K27me3 were enriched in the promoter region of *ANGPTL4* genes. Importantly, after *PVT1* silencing using a siRNA, EZH2 enrichment and H3K27me3 levels were significantly decreased in the promoter regions of *ANGPTL4* (Fig. 6D).

### *ANGPTL4* expression levels are elevated in PE placental tissues

**Figure 7:**
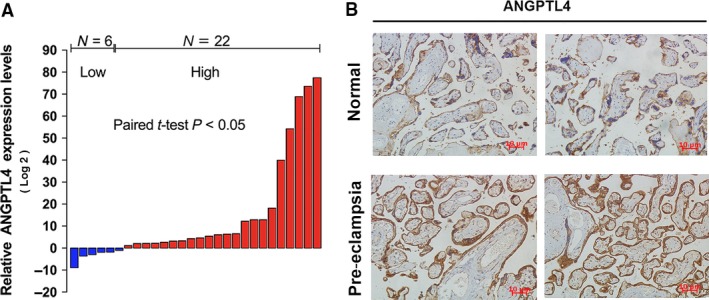
*ANGPTL4* expression in preeclampsia women. (**A**) Results are presented as the fold change in preeclampsia placental tissues relative to normal tissues, and *ANGPTL4* expression was classified into two groups (log2). (**B**) immunohistochemistry was performed to determine *ANGPTL4* protein abundance. *P < 0.05, **P < 0.01.

To establish the clinical significance of *ANGPTL4*, we first tested the expression levels of *ANGPTL4* mRNAs in placental tissues from women with severe PE and normal pregnancies. The results indicated that *ANGPTL4* mRNAs were up‐regulated in placental tissues from women with severe PE (Fig. 7A). In addition, immunohistochemistry showed that *ANGPTL4* protein levels were increased in placental tissues from women with severe PE (Fig. 7B). Taken together, our findings confirmed that *ANGPTL4* expression levels were inversely related to *PVT1* levels in placental tissues from patients with severe PE.

## Discussion

Gene expression profiling technologies and genome‐wide sequencing have recently been utilized to accelerate the identification of human genome transcripts. Approximately 90% of transcriptional products do not encode proteins 36; these transcripts are known as nonprotein‐coding RNAs (ncRNAs) and include microRNAs (miRNAs) and lncRNAs. Many previous studies have shown that lncRNAs are involved in various human disorders and cellular development 27, 37. Moreover, imbalance in lncRNAs affects essential cell biological processes, including cell proliferation, apoptosis 38, 39, 40, migration 40 and metastasis, *via* various molecular mechanisms 41, 42. We observed roles of maternally expressed 3 (MEG3) and sprouty RTK signalling antagonist 4‐intronic transcript 1 (SPRY4‐IT1) in our previous studies evaluating the progression of PE 29, 30. Consequently, identification of PE‐associated lncRNAs and exploration of their biological functions and clinical significance may provide insights into the improvement of lncRNA‐based diagnosis and prognosis in PE. However, the biological behaviour and molecular mechanisms of these lncRNAs in the pathogenesis of PE are still unclear.

*PVT1* has been explored in numerous pathological processes, including colorectal cancer 43, diabetic nephropathy 44 and gastric cancer 33. In the present study, we found that the relative *PVT1* levels were dramatically reduced in placental samples from women with PE compared with that in the control. Moreover, knockdown of *PVT1* inhibited HTR/SVneo cell proliferation and promoted apoptosis *in vitro*; conversely, overexpression of *PVT1* promoted proliferation. Silencing of *PVT1* expression stimulated G_0_/G_1_ phase accumulation and S phase reduction; however, *PVT1* overexpression promoted the progression of the trophoblast cell cycle. Together, our findings imply that *PVT1* might play an indispensable role in the proliferation and apoptosis of trophoblasts in PE and that *PVT1* might be a promising marker for foreseeing PE.

In this study, we found that *PVT1* was predominantly localized in the nucleus, indicating that PVT1 may affect transcriptional regulation. LncRNAs, which serve as molecular bait, can indirectly exert biological functions by binding to or titrating away RNAs or proteins, such as growth arrest and DNA‐damage inducible, alpha 45 and WD repeat domain 5 26. Among these RBPs, approximately 20% of all human lncRNAs bind to PRC2 to regulate downstream targets; 32 the PRC2 complex can mediate gene silencing by catalysing the trimethylation of H3K27. Our results showed that endogenous *PVT1* can interact with EZH2 protein in HTR/SVneo and JEG‐3 cells. Thus, *PVT1* may modulate PRC2‐mediated epigenetic regulation to affect the function of trophoblast cells and then may contribute to the occurrence and progression of PE.

Through a combination of RNA transcriptome sequencing and qRT‐PCR, we found that angiopoietin‐like 4 (*ANGPTL4)*, which has been reported to be related to cell cycle and apoptosis 46 was markedly up‐regulated after *PVT1* knockdown. *ANGPTL4,* a protein‐coding RNA gene located on chromosome 19p13.2, was identified to encode an adipokine involved in multiple biological processes such as cell progression and metastasis. However, the function of *ANGPTL4* in preeclampsia has not been studied. Here, we showed that EZH2 silenced *ANGPTL4* expression by epigenetic regulation. Thus, we propose that *PVT1* may exert its functions in part by binding to PRC2 and inhibiting *ANGPTL4* expression in trophoblasts in the context of PE.

In summary, we showed that *PVT1* was down‐regulated in clinical samples from patients with PE and that down‐regulation of this lncRNA may be associated with the pathogenesis of PE. Moreover, our study provides insights into the role of *PVT1* as a participant of the PRC2‐mediated epigenetic regulatory pathway in PE and as a novel indication for early diagnosis and treatment of PE. Further studies are required to illuminate other potential mechanisms through which *PVT1* participates in the biological functions of trophoblasts in PE.

## Conflicts of interest

No conflicts of interest were stated by authors.

## Supporting information


**Figure S1** The relative *PVT1* expression was detected by qPCR after treating with pcDNA‐*PVT1* or si‐RNAs, At least three times of biological replicates have been performed and presented. (Values are mean ± S.E.M.; **P < 0.01).

**Table S1** Sequence of primers and siRNAs.

**Table S2** Analysis of the RNA transcriptome sequencing data.
