# Supplementary figures and images for: The long non‐coding RNA PVT1 represses ANGPTL4 transcription through binding with EZH2 in trophoblast cell
Source: J Cell Mol Med. 2017 Nov 29;22(2):1272–82. doi: 10.1111/jcmm.13405 (PMC5783862; doi:10.1111/jcmm.13405)

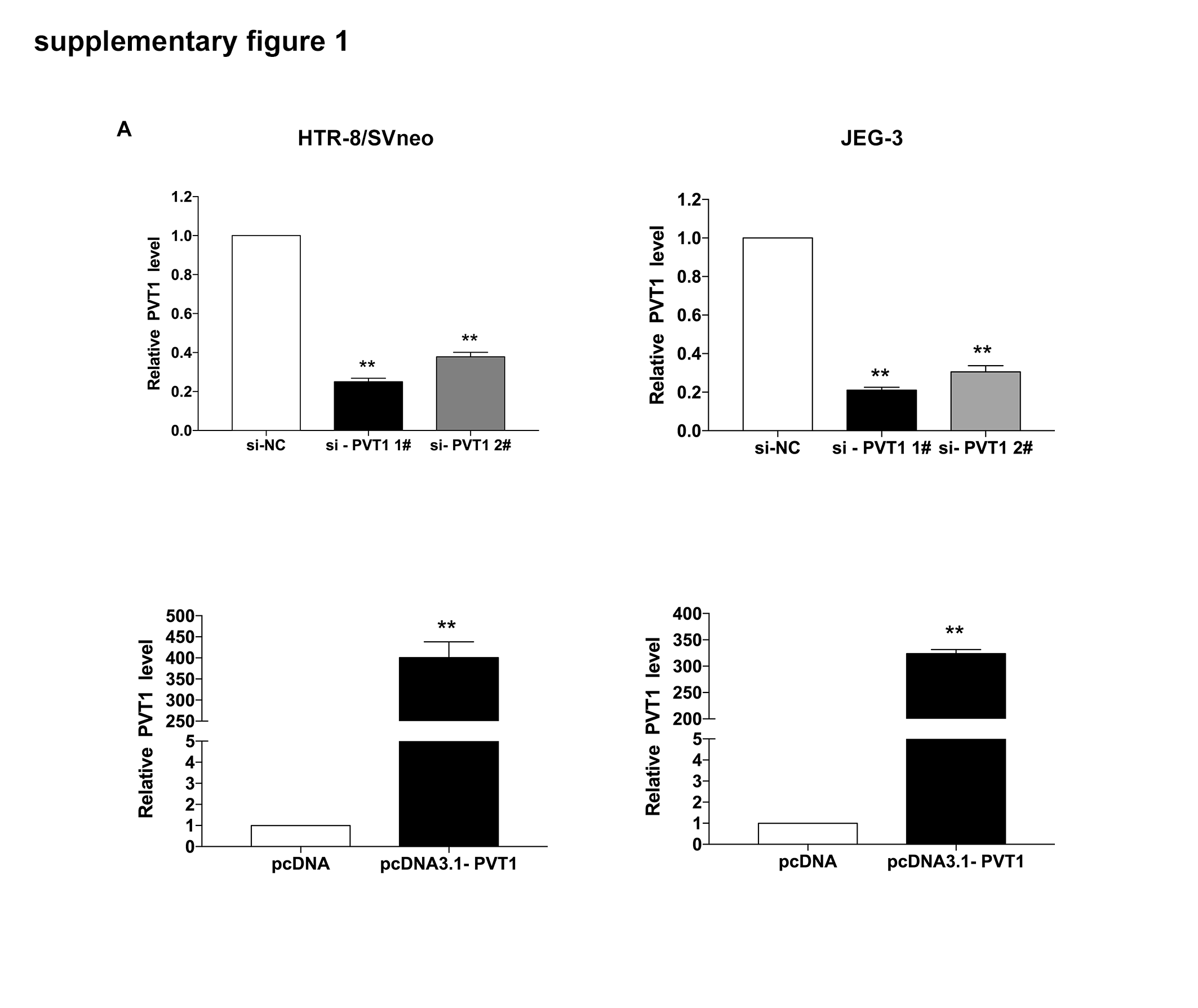

Supplement: Supplementary file 1 — Figure S1 The relative PVT1 expression was detected by qPCR after treating with pcDNA‐PVT1 or si‐RNAs, At least three times of biological replicates have been performed and presented. (Values are mean ± S.E.M.; **P < 0.01). [file JCMM-22-1272-s001.tif]
